# Role of C-terminal negative charges and tyrosine residues in fibril formation of α-synuclein

**DOI:** 10.1002/brb3.86

**Published:** 2012-08-10

**Authors:** Yasutaka Izawa, Hironobu Tateno, Hiroshi Kameda, Kazuya Hirakawa, Keiko Hato, Hisashi Yagi, Kunihiro Hongo, Tomohiro Mizobata, Yasushi Kawata

**Affiliations:** 1 Department of Biomedical Science, Institute of Regenerative Medicine and Biofunction, Graduate School of Medical Science, Tottori UniversityTottori, 680-8552, Japan; 2 Department of Chemistry and Biotechnology, Graduate School of Engineering, Tottori UniversityTottori, 680-8552, Japan

**Keywords:** Amyloid, amyloid formation mechanism, Parkinson's disease, protein aggregation, site-directed mutagenesis, α-Synuclein

## Abstract

α-Synuclein (140 amino acids), one of the causative proteins of Parkinson's disease, forms amyloid fibrils in brain neuronal cells. In order to further explore the contributions of the C-terminal region of α-synuclein in fibril formation and also to understand the overall mechanism of fibril formation, we reduced the number of negatively charged residues in the C-terminal region using mutagenesis. Mutants with negative charges deleted displayed accelerated fibril formation compared with wild-type α-synuclein, demonstrating that negative charges located in the C-terminal region of α-synuclein modulate fibril formation. Additionally, when tyrosine residues located at position 125, 133, and 136 in the C-terminal region were changed to alanine residue(s), we found that all mutants containing the Tyr136Ala mutation showed delays in fibril formation compared with wild type. Mutation of Tyr136 to various amino acids revealed that aromatic residues located at this position act favorably toward fibril formation. In mutants where charge neutralization and tyrosine substitution were combined, we found that these two factors influence fibril formation in complex fashion. These findings highlight the importance of negative charges and aromatic side chains in the C-terminal region of α-synuclein in fibril formation.

Synucleopathies make up a group of neurodegenerative disorders sharing in common the presence of intracellular inclusions comprised predominantly of α-synuclein (α-syn) amyloidogenic fibrils (Goedert 2001; Selkoe 2003; Shastry 2003; Norris et al. 2004). These neuronal α-syn inclusions, termed Lewy bodies and Lewy neurites, comprise one of the defining characteristics of Parkinson's disease and dementia with Lewy bodies (Spillantini et al. 1997; Baba et al. 1998; Goedert 2001; Selkoe 2003; Shastry 2003; Norris et al. 2004). Furthermore, the presence of brain α-syn inclusions is associated with many other neurodegenerative diseases (Goedert 2001; Norris et al. 2004).

α-Syn is a 140-amino acid, intrinsically disordered protein (Weinreb et al. 1996) that exists abundantly in neuronal cells (Bisaglia et al. 2009), where it is localized in the proximity of vesicles within presynaptic terminals (Nakajo et al. 1994; Iwai et al. 1995), although its actual physiological role is not well understood. The primary sequence of α-syn (Fig. 1a) is subdivided into three main domains; an N-terminal region that contains seven imperfect KTKEGV sequence repeats, a middle hydrophobic region that contains the amyloidogenic NAC region (amino acids 61–95: non-amyloid β component of Alzheimer's disease amyloid), and a C-terminal region rich in acidic amino acids (Ueda et al. 1993). The NAC region was originally identified as a 35-amino acid fragment of α-syn isolated from the brain tissue of Alzheimer's disease patients. Recently, the residues of Ala76–Lys96 within this hydrophobic region have been reported to be essential for forming the core of α-syn fibrils (Yagi et al. 2010).

**Figure 1:**
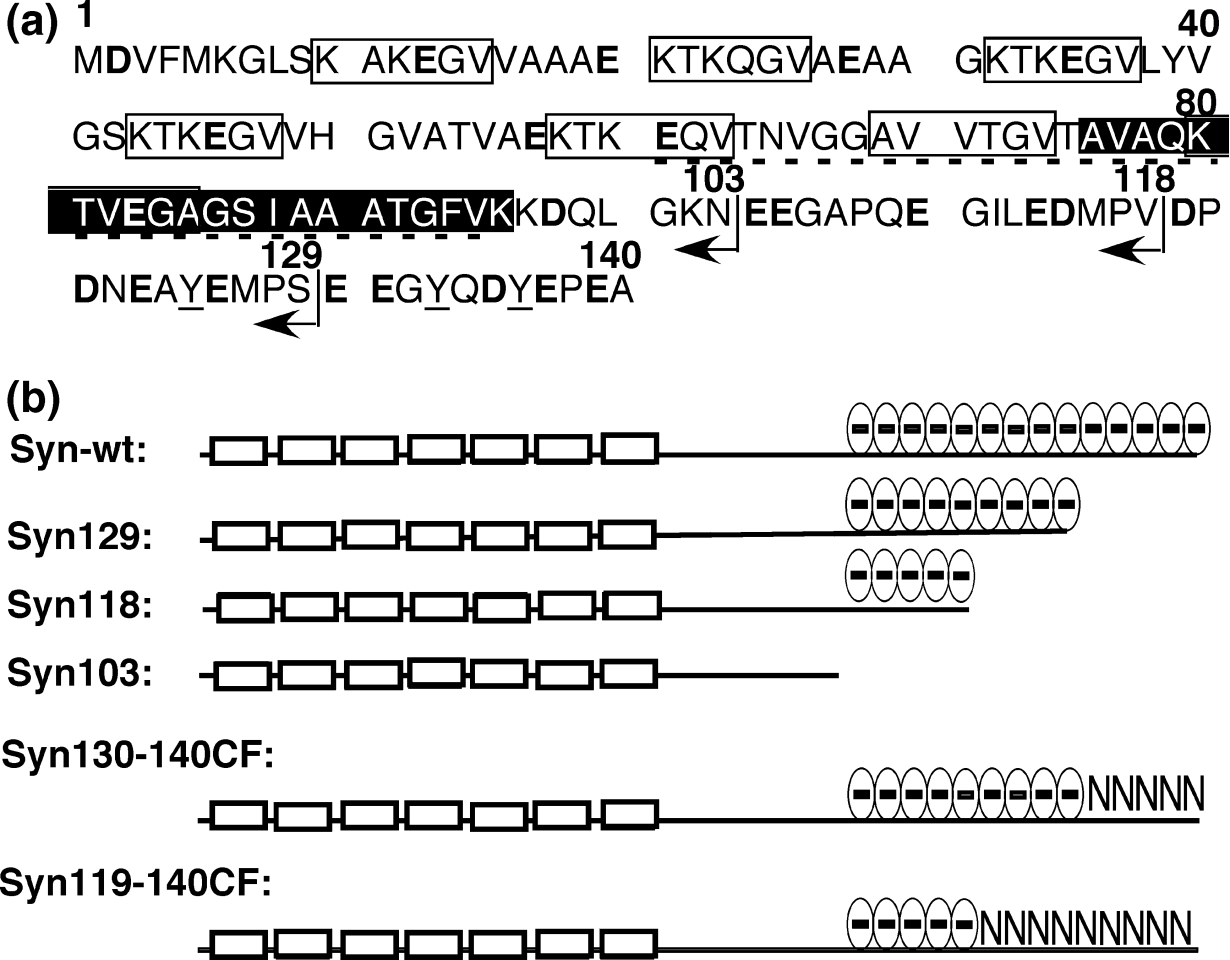
Amino acid sequence of α-syn and the schematic representation of mutants used in this study. (a) Amino acid sequence of α-syn. Open squares indicate the imperfect KTKEGV repeats. The closed square indicates the fibril core region determined previously. The NAC region is indicated by a dotted line under the sequence. Acidic amino acid residues are represented in bold and the tyrosine residues mutated in this study are underlined. The position of the C-terminal amino acid in the truncated mutants are denoted by arrows. (b) Schematic drawing of the relative polypeptide length and negative charge distributions of the C-terminal truncated or mutated proteins. The open boxes represent the imperfect KTKEGV region.

Amyloid fibrils that are generated during the course of various amyloidopathies share common structural characteristics: linear and twisted fibers with diameters of around 10–20 nm and with extensive cross-β secondary structure (Tan and Pepys 1994; Carrell and Lomas 1997; Sunde and Blake 1997; Sipe and Cohen 2000). Several studies indicate that the polymerization of α-syn progresses from disordered monomers to partially folded intermediates, which then form a “fibril nucleus,” and oligomers or protofibrils are assembled from these nuclei to finally elongate into “mature” amyloid filaments (Conway et al. 2000; Uversky et al. 2001; Yagi et al. 2005). This conversion of α-syn from monomer to amyloid fibrils is associated with a critical conformational change from an extended random coil to a compact predominantly β-pleated sheet (Serpell et al. 2000; Uversky et al. 2001; Li et al. 2002; Yagi et al. 2005). The molecular compaction that accompanies this conformational change is presumably important for nucleus formation (Higurashi et al. 2005). The conformational compaction has been reported to be correlated with elimination of electrostatic repulsion, by neutralization of the negatively charged side chains in the C-terminal region; in the presence of NaCl (Yagi et al. 2005) or at low pH (Cho et al. 2009; McClendon et al. 2009; Wu et al. 2009), the molecular size of α-syn decreases and fibril formation is accelerated remarkably. Furthermore, it is reported that tyrosine residues, which are also mostly located in the C-terminal region, are also related to fibril formation of α-syn, as fibril formation was inhibited or slowed in vitro by stabilization of off-pathway oligomers via nitration of tyrosine (Yamin et al. 2003) or by changing tyrosine residues to alanine (Ulrih et al. 2008). It is also reported that an increased tendency to form fibrils was observed for C-terminal truncated mutants of α-syn both in vitro (Crowther et al. 1998; Murray et al. 2003; Hoyer et al. 2004; Levitan et al. 2011) and in vivo (Li et al. 2005; Liu et al. 2005). About 10–30% of α-syn found in Lewy bodies is truncated at the C-terminal (Li et al. 2005). These findings suggest that the C-terminal region of α-syn is important for fibril formation, and hence, further study of this region is useful for understanding the steps leading to the onset of Parkinson's disease.

In this study, in order to further explore the role of C-terminal region of α-syn, we probed the relative contributions of negatively charged amino acid side chains and the tyrosine residues in fibril nucleus formation and elongation. Confirming earlier studies, various deletion mutants of α-syn readily formed amyloid fibrils compared with the wild-type α-syn (Syn-wt). The specific contribution of negatively charged side chains was determined by neutralizing these charges through Asp/Glu to Asn substitutions. We found that negatively charged side chains located in the C-terminal region of α-syn act to retard fibril formation. On the other hand, a specific tyrosine residue, Tyr136, displayed an active role in promoting α-syn fibrillation, as demonstrated in various Tyr136Ala mutations of α-syn and derivatives. Furthermore, mutation of Tyr136 to various other amino acids revealed that aromatic residues located at this position promote fibril formation. Finally, in mutants that combined both charge neutralization and tyrosine substitution, we found that these two modulating factors acted mostly independently in influencing fibril formation, with one glaring exception. This exception served to highlight an additional level of complexity in the fibril formation process of α-syn.

## Materials and Methods

### Expression and preparation of wild-type and mutant proteins of α-syn

The human α-syn gene was cloned into pET vector to make pET-SYN plasmid and expressed in *Escherichia coli* BLR(DE3) (Novagen, Darmstadt, Germany), and Syn-wt was purified as described previously (Yagi et al. 2005). C-terminal truncated or altered mutants of α-syn were constructed by using the QuikChange site-directed mutagenesis kit (Stratagene, Santa Clara, California), using pET-SYN as the template. Amino acid sequences of the C-terminal region of all α-syn proteins used in this study are summarized in Table 1. The successful construction of each mutant was confirmed by DNA sequence analysis of the entire α-syn coding region, and protein expression was checked by SDS–PAGE (12.5% polyacrylamide gel).

**Table 1:** Amino acid sequence of C-terminal region of syn-wt and truncated or mutated mutants

Syn-wt (ε^0.1%^_280 nm_ = 0.354)	…GKNEEGAPQEGILEDMPVDPDNEAYEMPSEEGYQDYEPEA – 140
Syn129 (0.227)	…GKNEEGAPQEGILEDMPVDPDNEAYEMPS – 129
Syn118 (0.125)	…GKNEEGAPQEGILEDMPV – 118
Syn103 (0.145)	…GKN – 103
Syn130-140CF (0.356)	…GKNEEGAPQEGILEDMPVDPDNEAYEMPSNNGYQNYNPNA – 140
Syn119-140CF (0.357)	…GKNEEGAPQEGILEDMPVNPNNNAYNMPSNNGYQNYNPNA – 140
Syn130-140CF/Y136A (0.310)	…GKNEEGAPQEGILEDMPVDPDNEAYEMPSNNGYQNANPNA – 140
Syn119-140CF/Y136A (0.310)	…GKNEEGAPQEGILEDMPVNPNNNAYNMPSNNGYQNANPNA – 140

SynY125/133/136A (0.105)	…GKNEEGAPQEGILEDMPVDPDNEAAEMPSEEGAQDAEPEA – 140
SynY125/133A (0.209)	…GKNEEGAPQEGILEDMPVDPDNEAAEMPSEEGAQDYEPEA – 140
SynY125/136A (0.209)	…GKNEEGAPQEGILEDMPVDPDNEAAEMPSEEGYQDAEPEA – 140
SynY133/136A (0.209)	…GKNEEGAPQEGILEDMPVDPDNEAAEMPSEEGAQDAEPEA – 140
SynY125A (0.311)	…GKNEEGAPQEGILEDMPVDPDNEAAEMPSEEGYQDYEPEA – 140
SynY133A (0.311)	…GKNEEGAPQEGILEDMPVDPDNEAYEMPSEEGAQDYEPEA – 140
SynY136A (0.311)	…GKNEEGAPQEGILEDMPVDPDNEAYEMPSEEGYQDAEPEA – 140

SynY136W (0.688)	…GKNEEGAPQEGILEDMPVDPDNEAYEMPSEEGYQDWEPEA – 140
SynY136F (0.309)	…GKNEEGAPQEGILEDMPVDPDNEAYEMPSEEGYQDFEPEA – 140
SynY136L (0.310)	…GKNEEGAPQEGILEDMPVDPDNEAYEMPSEEGYQDLEPEA – 140
SynY136S (0.311)	…GKNEEGAPQEGILEDMPVDPDNEAYEMPSEEGYQDSEPEA – 140
SynY136E (0.309)	…GKNEEGAPQEGILEDMPVDPDNEAYEMPSEEGYQDEEPEA – 140

*Upper panel*, The C-terminal amino acid sequence of Syn-wt and truncated or mutated mutants of acidic residues to Asn residue (marked by an underline). Syn130-140CF/Y136A and Syn119-140CF/Y136A are Tyr136 to Ala mutants of Syn130-140CF and Syn119-140CF, respectively. *Middle panel*, The C-terminal amino acid sequences of mutants, whose Tyr125, 133, 136 residue(s) were changed to Ala residue (marked by an underline). *Lower panel*, The C-terminal amino acid sequences of mutants, whose Tyr136 residue was changed to various different characteristic residues (marked by an underline).

All mutant proteins were expressed in *E. coli* BLR(DE3) and purified according to the method for Syn-wt (Yagi et al. 2005), with the exception of Syn118, Syn103, Syn119-140CF, and the Syn119-140CF/Y136A mutant. Syn118, Syn119-140CF, and Syn119-140CF/Y136A were purified utilizing Q-Sepharose anion-exchange chromatography at a pH different from the wild type (8.5 instead of 7.5). Regarding Syn103, due to the replacement of acidic amino acids in the C-terminal region, this mutant was unable to bind to Q-Sepharose. Syn103 was therefore purified as follows: After ultrasonic disruption, removal of nucleic acids by addition of streptomycin, heat-treatments, and fractionation by ammonium sulfate, protein solution desalting with a Cellulofine CH-25m (Seikagaku Kogyo, Tokyo, Japan) gel-filtration column, which had been equilibrated with purification buffer (50 mmol/L Tris–HCl, pH 7.5, containing 1 mmol/L EDTA, 0.1 mmol/L dithiothreitol, and 0.1 mmol/L phenylmethylsulfonyl fluoride). Protein fractions were pooled and loaded onto an SP-Sepharose cation-exchange column (GE Healthcare Life Sciences, Chalfront St. Giles, U.K.), which had been equilibrated with the purification buffer and eluted with a linear salt gradient (0–1 mol/L NaCl) at a flow rate of 1 mL/min. The purified Syn103 protein was desalted using a gel-filtration column equilibrated with 2.5 mmol/L ammonium bicarbonate. The three C-terminal truncation mutants, Syn130-140CF, and Syn119-140CF were lyophilized and stocked at 4°C until use. Protein concentrations of Syn-wt was determined by using a molar absorption coefficient of ε ^0.1%^_280 nm_ = 0.354 (Narhi et al. 1999), and protein concentrations of the other mutant proteins were determined by using individual calculated absorption coefficients (see Table 1) estimated from amino acid content (Pace et al. 1995).

### Amyloid fibril formation and ThioT binding assay

α-syn proteins (1 mg/mL) were induced to form fibrils at 37°C in fibrillation buffer (25 mmol/L Tris–HCl buffer, pH 7.5), containing either 0, 0.15, or 1 mol/L NaCl by linear (back and forth) shaking at a rate of 170 repetitions/min. Fibril formation was monitored by ThioT (Wako, Osaka, Japan) binding assay, using a HITACHI F-4500 (Hitachi Hightechnologies, Tokyo, Japan) or JASCO FP-6300 (JASCO, Tokyo, Japan) fluorescence spectrophotometer. Fibril samples were mixed with 25 μmol/L ThioT in phosphate-buffered saline, and fluorescence intensity was monitored at 480 nm upon excitation at 440 nm. In certain experiments, to boost the sensitivity as well as to facilitate fibrillation, a multiwell plate reader, ARVO X4 (Perkin Elmer, Waltham, Massachusetts), was used in measurements. One hundred fifty microliter aliquots of 1 mg/mL α-syn proteins dissolved in fibrillation buffer containing 20 μmol/L ThioT were pipetted into black multiwell plates (8 × 12-well plate; Greiner, Kremsmuenster, Austria) and sealed with a thermal seal. This multiwell plate was incubated in the plate reader by linear shaking (5 mm back and forth) at 37°C. At appropriate intervals, fibril formation was monitored by measuring the ThioT fluorescence of each well through the transparent quartz floor of the plate (“bottom-read method”; measurement time of 0.1 sec: excitation, 440 nm; emission, 486 nm). Two independent experiments were performed and data obtained were averaged.

In order to obtain stable and reliable data, especially for experiments involving Tyr substitution mutants, freshly purified mutant proteins were immediately used in amyloid fibril formation experiments in fibrillation buffer containing 1 mol/L NaCl without prior lyophilization and storage. For variants of α-syn that were lyophilized and stored for certain amounts of time, samples were dissolved in 6 mol/L guanidine hydrochloride, incubated for 30 min at 25°C, and then desalted and changed to amyloid fibril formation buffer with PD-10 column immediately before fibril formation experiments.

### TEM and AFM measurements of amyloid fibrils

TEM measurements were performed on a JEOL-100CX (JEOL, Tokyo, Japan) transmission electron microscope operated at 80 kV. Samples were diluted 10-fold with water and negatively stained with 2% (w/v) uranyl acetate solution on copper grids (400-mesh) covered by carbon-coated collodion film (Nisshin EM, Tokyo, Japan). Observation magnification was 9400–34,000. AFM measurements were performed using a Digital Instruments Nanoscope IV scanning microscope (model MMAFM-2) at 25°C. Measurements were performed using air tapping mode. Fifteen microliters of 10-fold diluted fibril solution was put onto freshly cleaved mica, incubated for 30 min at room temperature, and then washed with 150 μL of water and dried.

### CD measurements

CD spectra were measured using a Jasco J-720 spectropolarimeter equipped with a constant temperature cell holder at 25°C. Far-UV CD spectra were recorded using 1-mm light path cells. Samples were measured at a protein concentration of 0.35 mg/mL.

## Results

### Effects of negative charges in the C-terminal region on fibril formation of α-syn

**Figure 2:**
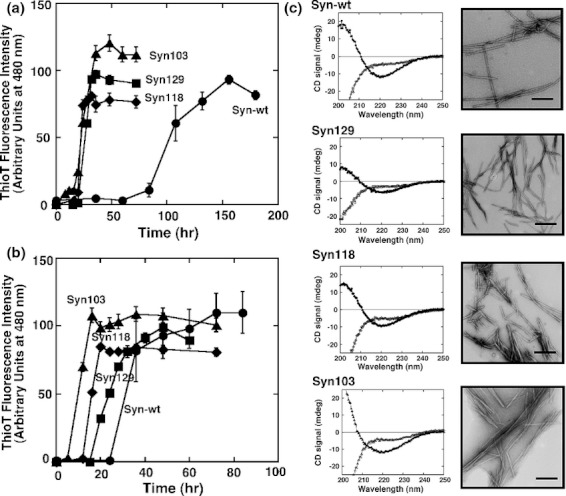
Fibril formation characteristics of the C-terminal truncated mutants. (a) and (b) Amyloid fibril formation monitored by ThioT binding assay. Conditions were 1 mg/mL protein in 25 mmol/L Tris–HCl buffer, pH 7.5, containing 0 mol/L (a) and 150 mmol/L (b) NaCl, at 37°C. Closed circles indicate Syn-wt; closed squares, Syn129; closed diamonds, Syn118; closed triangles, Syn103. Standard error bars derived from at least three independent measurements are also shown. (c) CD spectra and TEM images of Syn-wt, Syn129, Syn118, and Syn103. In the CD spectra, open and closed symbols represent for samples before and after incubation for fibril formation, respectively. The scale bar in each panel of TEM image represents 200 nm.

In order to explore the role of negative charges in the C-terminal region of α-syn, we prepared various C-terminal truncated mutants and examined the effects of each mutation on fibril formation. As shown in Figure 1 and Table 1, mutants Syn129, Syn118, and Syn103 were prepared, where 11, 22, and 37 residues were, respectively, deleted from the C-terminus. α-syn has 14 amino acids that are negatively charged under physiological conditions in the C-terminal region spanning positions 104 and 140. Syn129 still retains 10 negatively charged amino acids, Syn118 has five, and Syn103 has none of the original 14 negatively charged amino acids. These C-terminal truncated mutants were examined for the ability to form fibrils. We first performed experiments using buffer with no NaCl added in order to assess the effects of negative charge truncation sensitively. As shown in Figure 2a, whereas Syn-wt formed amyloid fibrils after an 80-h incubation, all C-terminal truncated mutants formed fibrils much more quickly (∼10–20 h), suggesting that deletion of negative charges from the C-terminal region caused a significant change in the fibril formation rate under these conditions; a significant acceleration in fibril formation was observed even for Syn129, which had only four acidic amino acid residues deleted from the C-terminus.

To observe in more detail differences caused by deleting some or all of the acidic amino acid residues from the α-syn C-terminus, fibril formation experiments were performed in buffer containing 150 mmol/L NaCl, a salt concentration that more closely resembles physiological conditions. At 150 mmol/L NaCl, as shown in Figure 2b, Syn103 formed fibrils most quickly, and Syn-wt was the slowest, although a large acceleration was observed for Syn-wt when compared to the reaction in 0 mol/L NaCl (from 80 to 25 h). In the presence of 150 mmol/L NaCl (Fig. 2b), the degree of acceleration correlated very well with the number of negative residues deleted from the C-terminus. A similar correlation has been reported by Hoyer et al. (2004). Notably, as the rate of fibril extension (as monitored by the slope of ThioT fluorescence increase seen after the initial lag stage) was similar for all of the mutants and Syn-wt (Fig. 2b), it is likely that the removal of negative charges from the C-terminal region of α-syn mainly affects fibril nucleus formation. This correlation was also reported by Levitan et al. (2011) very recently. In Figure 2c, CD spectra and TEM images of Syn-wt and all mutant proteins after formation of fibrils are shown. In CD spectra, β-sheet-like structural characteristics were observed for all proteins. In TEM images, although minute differences in morphology were observed depending on the mutant protein, linear fibers with similar dimensions (∼20 nm in width) were observed.

**Figure 3:**
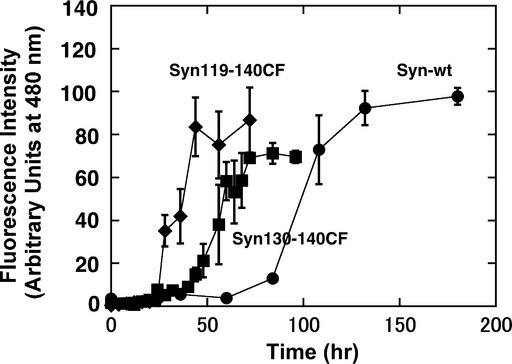
Fibril formation characteristics of the charge-free full-length α-syn mutants. Conditions were 1 mg/mL protein in 25 mmol/L Tris–HCl buffer, pH 7.5 at 37°C. Closed circles indicate Syn-wt; closed squares, Syn130-140CF; closed diamonds, Syn119-140CF. Standard error bars derived from at least three independent measurements are also shown.

In order to clarify the notion that the acceleration of nucleus formation is due only to changes in negative charge, rather than overall polypeptide length, we next prepared two charge-neutralized full-length α-syn mutants, Syn130-140CF and Syn119-140CF. Syn130-140CF is a full-length (140 AA) α-syn polypeptide in which four Glu residues and one Asp residue located between positions 130 and 140 have been replaced by the noncharged amino acid residue asparagine. In Syn119-140CF, six Glu and three Asp residues between positions 119 and 140 have been changed to Asn (Fig. 1 and Table 1). Fibril formation of these mutants were examined and compared with Syn-wt. As shown in Figure 3, fibril formation was accelerated to the order of ∼25 h for Syn119-140CF and ∼40 h for Syn130-140CF. When compared to Syn-wt (∼80 h), we observed that fibrillation was accelerated increasingly, depending on the number of negatively charged amino acid residues that were neutralized at the C-terminus. This finding served to isolate the effects of negative charge loss from the effects of polypeptide truncation, and show clearly that removal of negative charges from the C-terminal region of α-syn results in an accelerated formation of fibrils, mainly through facilitating fibril nucleus formation.

### Role of tyrosine residue in the C-terminal region of α-syn on the fibril formation

**Figure 4:**
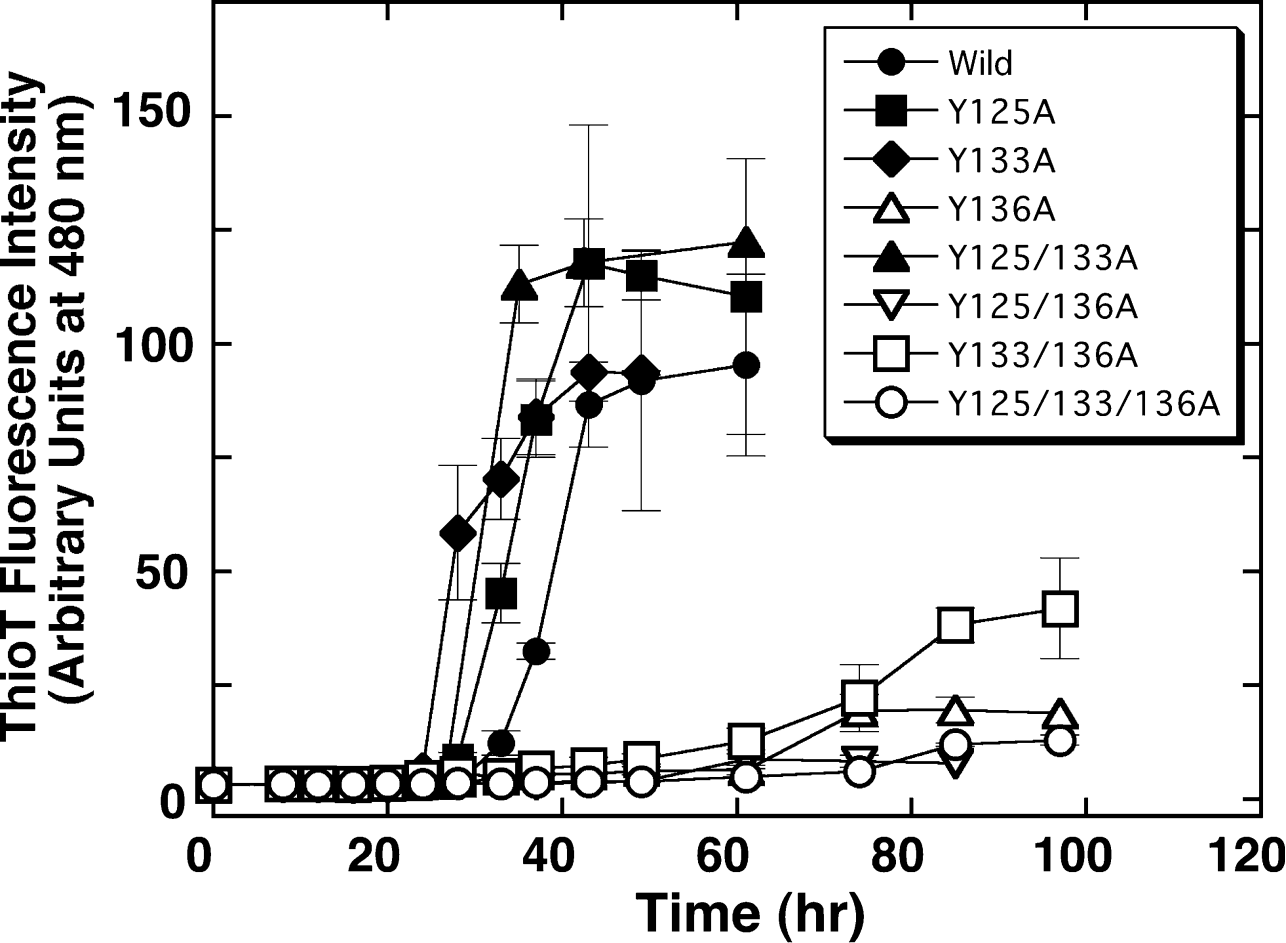
Fibril formation characteristics of various tyrosine substitution α-syn mutants. Conditions were 1 mg/ml protein in 25 mmol/L Tris–HCl buffer, containing 1 mol/L NaCl, pH 7.5 at 37°C. Representations of symbols are explained in the figure. Standard error bars derived from at least three independent measurements are also shown.

In the C-terminal region of α-syn, three tyrosine residues are also found at positions 125, 133, and 136. In order to understand the role of these tyrosine residues, we prepared several mutants where these tyrosines were selectively replaced with alanine residues, and examined the effects of these mutations on amyloid fibril formation. The mutants constructed are grouped into single-residue substitution mutants (SynY125A, SynY133A, and SynY136A), double-substitution mutants (SynY125/133A, SynY125/136A, and SynY133/136A), and a mutant with all three tyrosines replaced (SynY125/133/136A). As shown in Figure 4, these mutants could be functionally regrouped into two groups, i.e., one group consisting of SynY125A, SynY133A, and SynY125/133A, which displayed a similar lag (nucleation) time (25–30 h) and fibril extension rates similar to that of Syn-wt. The other group included SynY136A, SynY125/136A, SynY133/136A, and SynY125/133/136A, with a prolonged nucleation time (60–70 h) and a slower fibril extension rate compared with Syn-wt. The common characteristic of members of the latter group was mutation of the tyrosine residue at position 136 to alanine. These results suggested strongly that Tyr136 plays a critical role in the amyloid fibril formation mechanism of α-syn.

**Figure 5:**
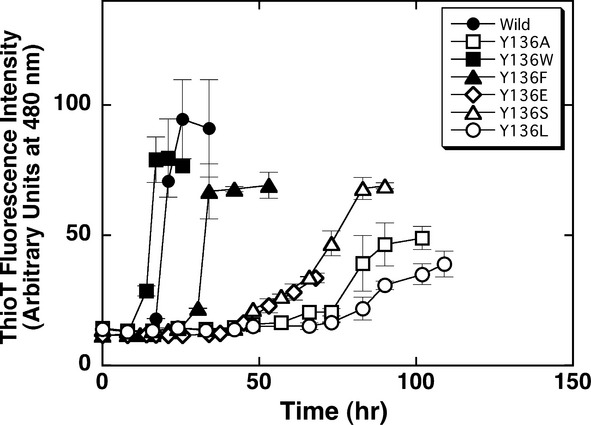
Fibril formation characteristics of various Tyr136 substitution α-syn mutants. Conditions were the same as described in the legend of Figure 4. Representations of symbols are explained in the figure. Standard error bars derived from at least three independent measurements are also shown.

In order to investigate further the role of Tyr136 in α-syn fibril formation, we replaced Tyr136 with various other amino acid residues. The mutants additionally prepared were substitutions to Trp, Phe, Leu, Ser, and Glu. Trp and Phe are hydrophobic and aromatic residues similar to Tyr, Leu is a residue with a hydrophobic aliphatic side chain, Ser is a hydrophilic residue without charge, and Glu represents a hydrophilic residue with charge. As shown in Figure 5, the behavior of SynY136W was almost the same as Syn-wt, and that of SynY136F was also similar to Syn-wt, although the lag time was slightly delayed. Fibril extension rates of these two proteins were almost the same as Syn-wt. In contrast, SynY136E, SynY136S, SynY136A, and SynY136L showed characteristics that were significantly different from Syn-wt, i.e., the nucleation times were prolonged and the fibril extension rates were clearly decreased compared to Syn-wt. In other words, aromatic residues at position 136 acted favorably toward both nucleus formation and fibril extension reactions, and hydrophilic or aliphatic residues at this position disfavored this reaction. These findings supported our proposal that Tyr136 of α-syn plays an important role in the fibril formation, during both fibril nucleation and fibril extension.

### Combining the Tyr136Ala mutation with charge neutralization in the C-terminal region

**Figure 6:**
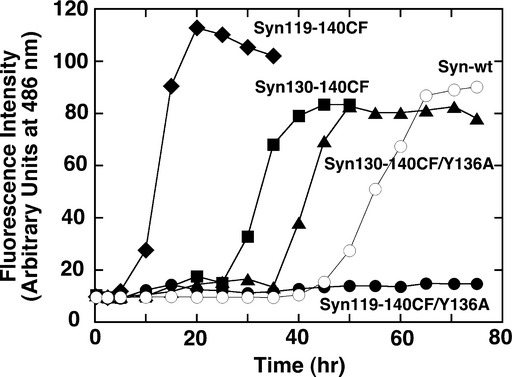
Fibril formation characteristics of Tyr136Ala mutants of Syn130-140CF and Syn119-140CF. Conditions were 1 mg/mL protein in 25 mmol/L Tris–HCl buffer, containing 150 mmol/L NaCl, pH 7.5 at 37°C. Plate readers of ARVO X4 (Perkin Elmer) was used for measurements by shaking. Representations of symbols: Syn-wt (open circles), Syn130-140CF (closed squares), Syn119-140CF (closed diamonds), Syn130-140CF/Y136A (closed triangles), Syn119-140CF/Y136A (closed circles).

As shown above, the deletion of negative charges in the C-terminal region accelerated fibril formation of α-syn (Fig. 3), and the presence of tyrosine 136 in the same region promotes fibril formation by allowing nucleus formation (Fig. 4. and 5). In order to evaluate the relationship between these two modulating factors of fibril formation at neutral pH (near physiological conditions), we prepared Tyr136Ala mutants of Syn130-140CF and Syn119-140CF (hereafter called Syn130-140CF/Y136A and Syn119-140CF/Y136A, respectively; Table 1). To enhance our ability to detect fibril formation at pH 7.5 in the presence of 150 mmol/L NaCl, we used a multiwell plate reader for measurements, and also used the mixing function of the reader to promote fibril formation. It should be noted, thus, that this fibril formation experiment was performed under conditions different from other experiments. As shown in Figure 6, Syn130-140CF and Syn119-140CF commenced fibril formation after a ∼25- and ∼5-h incubation, respectively, and both completed forming fibrils after a further 15-h incubation under these conditions. These reactions were accelerated compared to that (∼45 h) of wild-type α-syn (see open circles in Fig. 6), and a correlation was seen between the time necessary to complete fibril formation and the number of negative charges removed. When Tyr136 was changed to Ala in Syn119-140CF, fibril formation was greatly suppressed, indicating that the presence of Tyr138 was the predominant factor in the initiation of fibril formation that negates the effects of charge removal. This result is in overall agreement with the results shown in Figures 4 and 5. However, a most peculiar result was obtained when similar experiments were performed on the Syn130-140CF/Y136A mutant. In this mutant, the ability to form fibrils after a prolonged incubation was retained in spite of tyrosine substitution, although the time required for fibril completion was increased greatly compared with Syn130-140CF. In other words, in the Syn130-140CF/Y136A mutant, the fibril promoting effect, attributed to neutralization of negative charges, served to partially offset the strong fibril suppression caused by the Tyr-Ala substitution at position 136.

**Figure 7:**
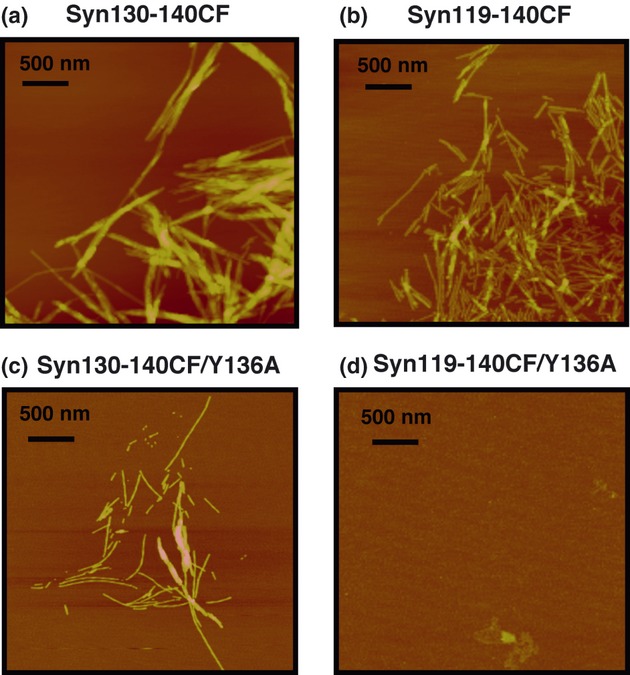
AFM images of α-syn mutants prepared in Figure 6. (A) Syn130-140CF, (B) Syn119-140CF, (C) Syn130-140CF/Y136A, and (D) Syn119-140C/Y136A. The scale bars represent 500 nm.

Could there be any morphological differences in the fibrils formed by these combination mutants that would explain this interesting result? To address this point, we took samples of the fibrils formed in Figure 6 and subjected them to AFM analysis. As shown in Figure 7, fibrils observed with AFM agreed well with the Thioflavin-T fibril profiles. A marked lack of distinct fibrils was detected in incubated samples of Syn119-140CF/Y136A, while fibril forms that tended to clump together were observed for Syn130-140CF and Syn119-140CF samples. Interestingly, in samples of Syn130-140CF/Y136A we also observed fibrils; however, the fibrils seemed morphologically distinct from the other two fibril samples we observed. This minor difference in fibril morphology may be a hint to the complex mechanism of fibrillation that is modulated by these mutations.

## Discussion

α-Syn is an intrinsically disordered protein expressed abundantly in neuronal cells (Weinreb et al. 1996; Bisaglia et al. 2009) and is regarded as being one of the causative proteins of Parkinson's disease (Spillantini et al. 1997; Baba et al. 1998; Goedert 2001; Selkoe 2003; Shastry 2003; Norris et al. 2004). To understand the fibril formation mechanism of α-syn is critical to developing a medical treatment for Parkinson's disease. In this study, we focused on the negative charges and tyrosine residues located in the C-terminal region of α-syn. Previous studies (Uversky et al. 2001; Yagi et al. 2005; Cho et al. 2009; McClendon et al. 2009; Wu et al. 2009) reported that the α-syn molecule forms compact molecular species at lower pH or in the presence of salts such as NaCl. Under such conditions, neutralization of the negative charges of Asp and Glu occur at the C-terminal region, and consequently electrostatic repulsion is reduced and the molecule is able to collapse. This compaction is important to both fibril nucleation, and subsequent fibril extension. This charge effect is also seen directly in deletion mutants of α-syn, i.e., an increased tendency to form fibrils is observed for C-terminal truncated mutants of α-syn both in vitro (Crowther et al. 1998; Murray et al. 2003; Levitan et al. 2011) and in vivo (Li et al. 2005; Liu et al. 2005).

Here, in addition to confirming the effects of C-terminal residue deletion on α-syn fibril formation (Fig. 1. and 2), we probed the contributions of electrostatic repulsion directly by constructing substitution mutants where C-terminal Asp and/or Glu residues were altered to Asn residues (Fig. 3). From the results, it was revealed clearly that the acceleration of fibril formation, mainly fibril nucleus formation is due mainly to the elimination of negative charges, but not to decreases in polypeptide length of the C-terminal region. This result also agrees well with the finding that the fibril formation of α-syn is accelerated by charge shielding through addition of NaCl (Yagi et al. 2005), MgCl_2_, or spermine (Hoyer et al. 2004), or by lowering the pH (Hoyer et al. 2004; Cho et al. 2009; McClendon et al. 2009; Wu et al. 2009). The importance of the C-terminal 40 amino acids for fibril formation is also reported by Horvath et al. (2012) very recently. They observed an accelerated fibril formation of α-syn in the presence of a dihydro thiazolo ring fused 2-pyridone derivative, and found through NMR experiments that the compound interacts with amino acid residues 1–100, while residues 101–140 remained flexible in solution. This result demonstrates that masking the N-terminal region of the α-syn polypeptide results in conditions favorable for fibril formation, most likely by increasing accessibility to important portions of the C-terminal region.

As the fibril nucleus is stabilized by oligomerization, electrostatic repulsion between molecules may exert an inhibitory effect during fibril nucleus formation. Recently, we determined the fibril nucleus core peptide region of α-syn as the segment corresponding to Ala76–Lys96 (Yagi et al. 2010). When the 14 negative charges located between positions 104 and 139, which are relatively close to this nucleus core region, are removed by either deletion or mutation, intermolecular interactions of the fibril nucleus peptide regions would be favored, which in turn would accelerate fibril nucleus formation. Even under conditions similar to the physiological salt concentration, the effects of negative charge were observed, as seen in Figure 2b, suggesting that this electrostatic contribution is significant.

In the C-terminal region of α-syn, we also find tyrosine residues at positions 125, 133, and 136. We have examined the relative contributions of these Tyr residues on fibril formation by mutating them to Ala residue(s) (Fig. 4), and found that Tyr136 was a critical element that promoted fibril formation. Simply changing Tyr136 to Ala was sufficient to significantly suppress both fibril nucleus formation (evidenced by the increased lag time) and fibril extension (seen by a decrease in the rate of fluorescence intensity increase). This Tyr could be replaced by Trp or Phe with minimal effects to the fibril formation mechanism, but not by Ser, Glu, or Leu (Fig. 5). These findings demonstrate that aromatic residues at position 136 are necessary for fibril formation. In other words, a possible way to suppress the fibril formation of α-syn may be to change Tyr136 to other nonaromatic amino acid residues.

Because the two factors that we focused upon in this study were located in the same C-terminal region of the α-syn polypeptide, we combined these two mutants to probe for any synergistic effects on fibril formation. Our results surprisingly pointed toward a very complex nucleation mechanism that dictated synuclein fibrillation. First of all, the relative importance of the tyrosine residue at position 136 was highlighted in our experiments. The results seen with the Syn119-140CF/Y136A mutant was a good example of the dominance of the tyrosine residue in dictating the formation of fibers (Fig. 6). However, if we refrained from neutralizing all of the negative charges in the C-terminal region, removing only the charges between residues 130 and 140, we observe that the absence of Tyr136 may be overcome, leading to fibrillation. This result is in apparent conflict with the dominant effects of tyrosine substitution seen in the other mutants probed in this study.

When we observed the shapes of the fibrils formed in Figure 6, we found that fibrils formed by Syn130-140CF/Y136A were slightly different from the other samples (Fig. 7). Perhaps another, alternate pathway of fibril formation that is accessible only to this mutant exists. This may be because retaining the negative charges between residues 119 and 129 allows access to a new site that promotes nucleation, perhaps due to differences in the overall secondary structure. In Syn119-140CF/Y136A, removal of all of the negative charges in this region may cause the alternate site to be occluded once more, resulting in the complete suppression of fibril formation brought about by the absence of Tyr 136.

Our results have revealed that there may be many pathways involving multiple factors in the C-terminal region that initiate the formation of α-syn fibrils, and further careful analysis is necessary to completely understand the process of fibril nucleation and extension. In this context, we feel it worthwhile to emphasize another experimental result that was reported by others and confirmed by us; that α-syn also shows an increased tendency to form fibrils when the C-terminal region of interest is completely removed (Fig. 2). A complete understanding of the process of α-syn fibril formation must therefore provide an understanding of all of these diverse facets of the initial steps of fibril formation.

**Figure 8:**
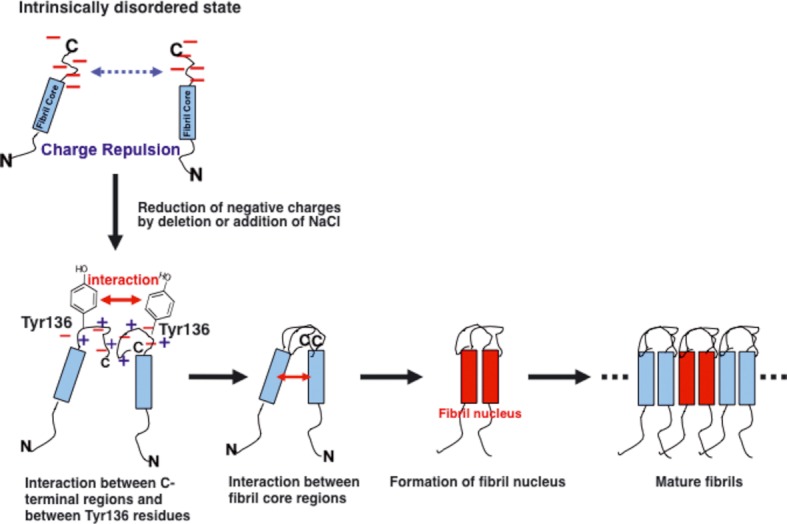
A schematic model of α-syn fibril formation mechanism. Roles of the C-terminal negative charges and Tyr136 on the fibril formation, especially on the fibril nucleus formation step, are shown. The long blue squares represent the fibril core region site of the α-syn (Yagi et al. 2010). See text for details.

We have attempted to figure out a possible mechanism of α-syn amyloid fibril formation that explains our findings. The schematic model is shown in Figure 8. α-Syn is intrinsically disordered and the polypeptide may assume an expanded conformation due to the repulsion of negative charges located in the C-terminal region, including other ensemble conformations (Heise et al. 2005). Also, charge repulsion should suppress various intermolecular interactions important to molecular association (Levitan et al. 2011). Upon deletion of the C-terminal negative charges or addition of NaCl, the electrostatic repulsion is reduced or shielded and intermolecular interactions centered upon this region is able to occur. Then, intermolecular interactions involving Tyr136 are initiated, probably due to the aromatic hydrophobic (Makin et al. 2005; Levy et al. 2006; Yagi et al. 2008, 2010) or π–π ring stacking interaction (Levy et al. 2006). The commitment of Tyr136 in this step is very important for fibril formation. From this increased intermolecular interaction, the fibril core region (Ala76–Lys96) (Yagi et al. 2010), which is relatively close to the C-terminal region, now begins to form the fibril nucleus. Once the fibril nucleus forms tightly, fibril extension reaction begins rapidly. During this extension step, Tyr136 also affects the fibril extension rate through aromatic ring interactions. For the C-terminal truncation mutants that lack both negative charges and Tyr136, fibrillation must wait until the hydrophobic characteristics of the fibril core region trigger molecular association. Thus, the negative charges and Tyr136 located in the C-terminal region of α-syn both play critical roles in the mechanism of amyloid fibril formation.

Finally, these findings in this study may shed light on the gradual and persistent fibrillation mechanism of this intrinsically disordered protein, and also may lead to the development of a medical treatment for Parkinson's disease. In our hands, a mutant α-syn in which the amino acid residues between 119 and 140 have been deleted (Syn118) readily forms fibrils. In contrast, Syn119-140CF/Y136A, where the relevant amino acids in the same sequence region (negatively charged residues, and Tyr136) have been substituted, is unable to form fibrils (Fig. 6). This comparison seems to suggest that the charge-neutralized, tyrosine-deleted C-terminal region of Syn119-140CF/Y136A may be actively inhibiting the fibril formation of α-syn, perhaps through intramolecular or intermolecular interaction with the fibril core sequence (residues Ala76–Lys96; Yagi et al. 2010). If true, a synthetic peptide corresponding to the C-terminal amino acid sequence of Syn119-140CF/Y136A might conceivably be utilized as an inhibitor of fibrillation, i.e., such peptide administered in vivo may interact with α-syn and prevent intermolecular interactions. Through utilization of this peptide, a new medical treatment for Parkinson's disease may eventually be developed.
